# Supplementary material for: Size-Controllable Nanosystem with Double Responsive for Deep Photodynamic Therapy
Source: Pharmaceutics. 2023 Mar 14;15(3):940. doi: 10.3390/pharmaceutics15030940 (PMC10056800; doi:10.3390/pharmaceutics15030940)
Supplement: Supplementary file 1 [file pharmaceutics-15-00940-s001.zip › pharmaceutics-2202444-supplementary.pdf]

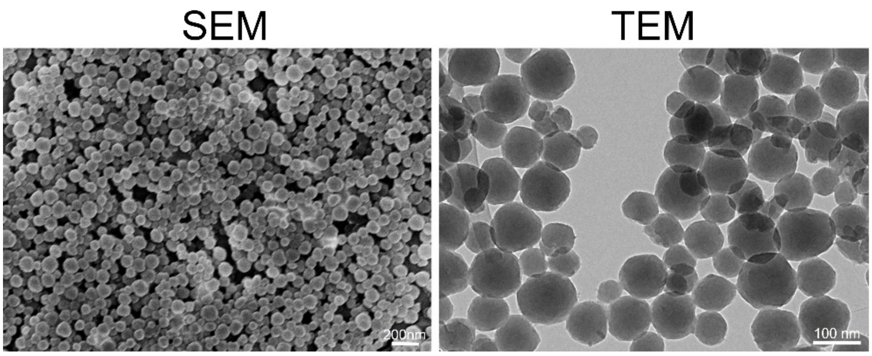

Figure S1. Electron microscope images of PCN nanoparticles by SEM and TEM.

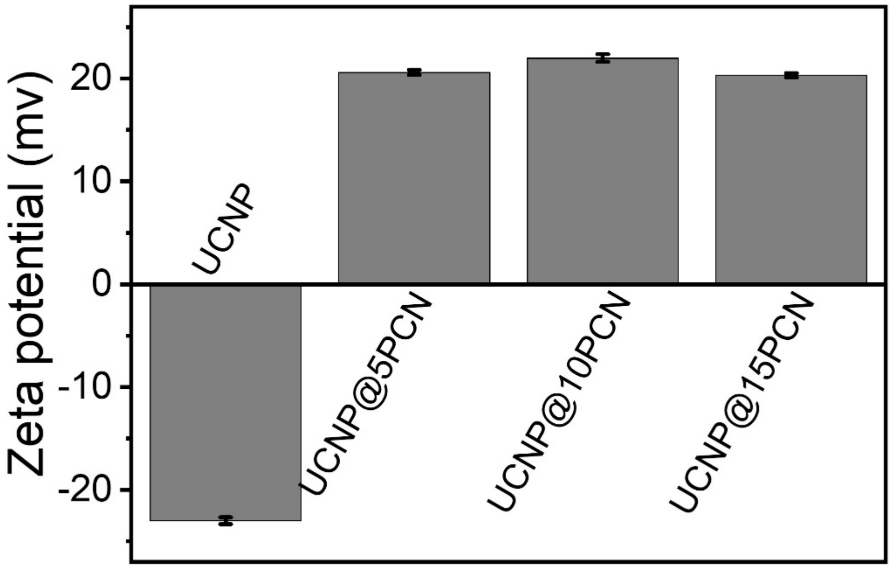

Figure S2. Zeta potential of different nanoparticles by DLS. Charge reversal of nanoparticles indicates successful encapsulation of PCN nanoparticles.

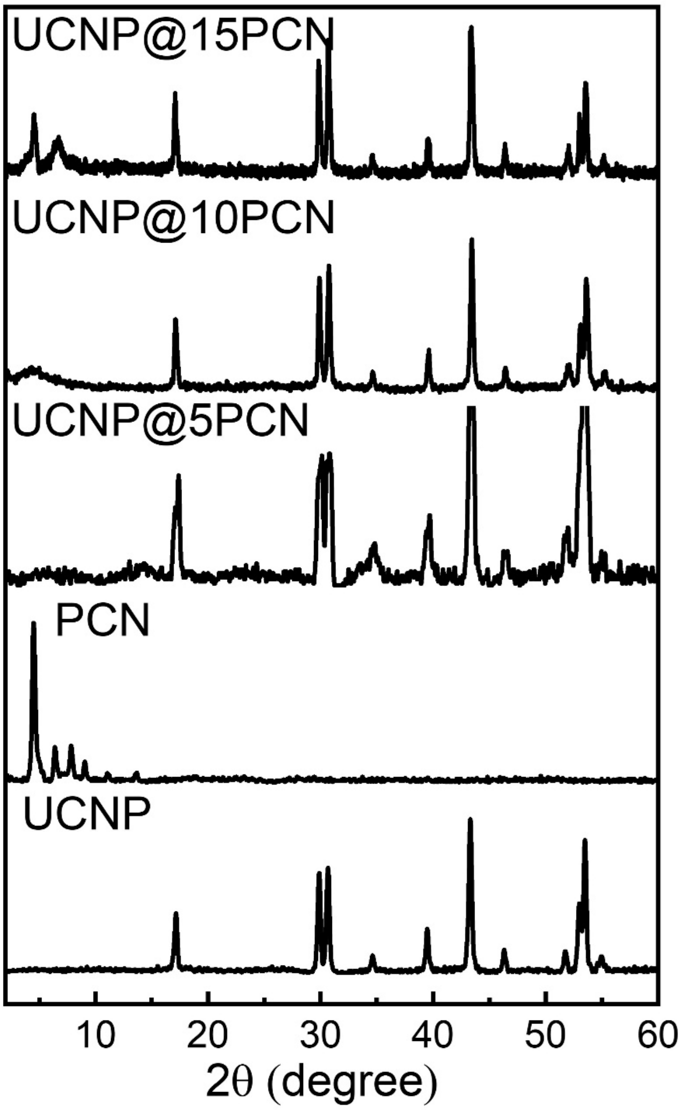

Figure S3. PXRD pattern of different nanoparticles. The characteristic peak of PCN increases with increasing thickness of PCN layer.

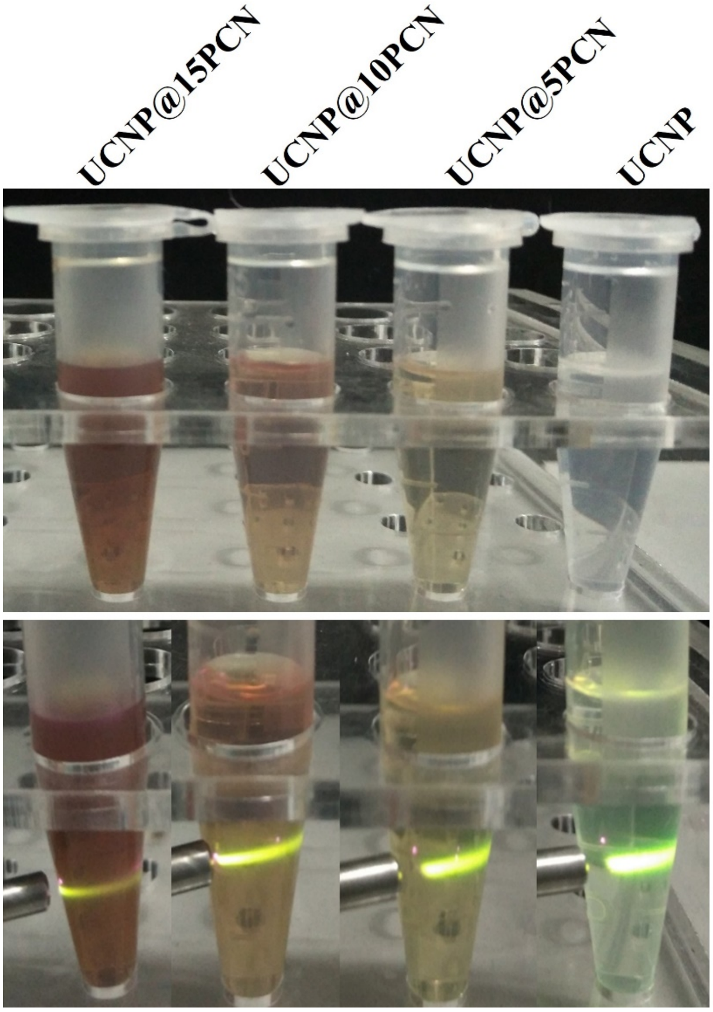

Figure S4. Images of different nanoparticles before and after 980 nm laser irradiation.

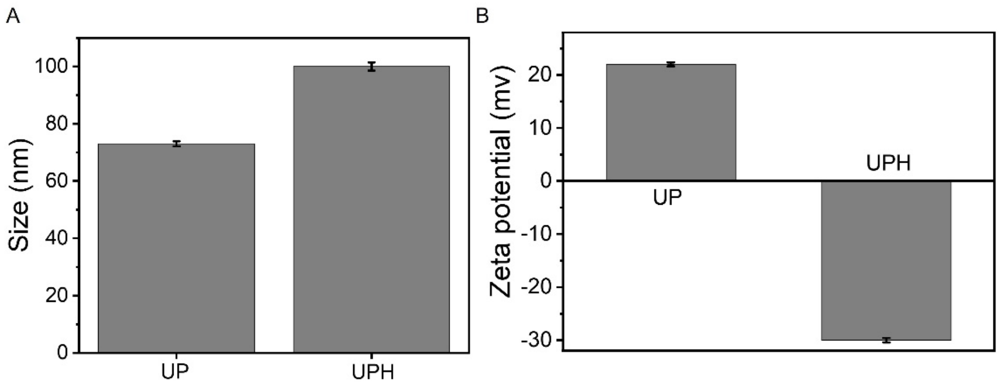

Figure S5. (A) Size and (B) zeta potential of UP and UPH nanoparticles by DLS.

Table 1. Comparison of the properties of nanoparticles with different thicknesses.

Sample	UCNP@5PCN	UCNP@10PCN	UCNP@15PCN
Size (nm)	65	73	80.3
Zeta Potential (mv)	20.6	22	20.32
Absorption	UCNP@5PCN< UCNP@10PCN<UCNP@15PCN		
Fluorescence intensity	UCNP@5PCN> UCNP@10PCN>UCNP@15PCN		
ROS generation	UCNP@5PCN< UCNP@10PCN>UCNP@15PCN		

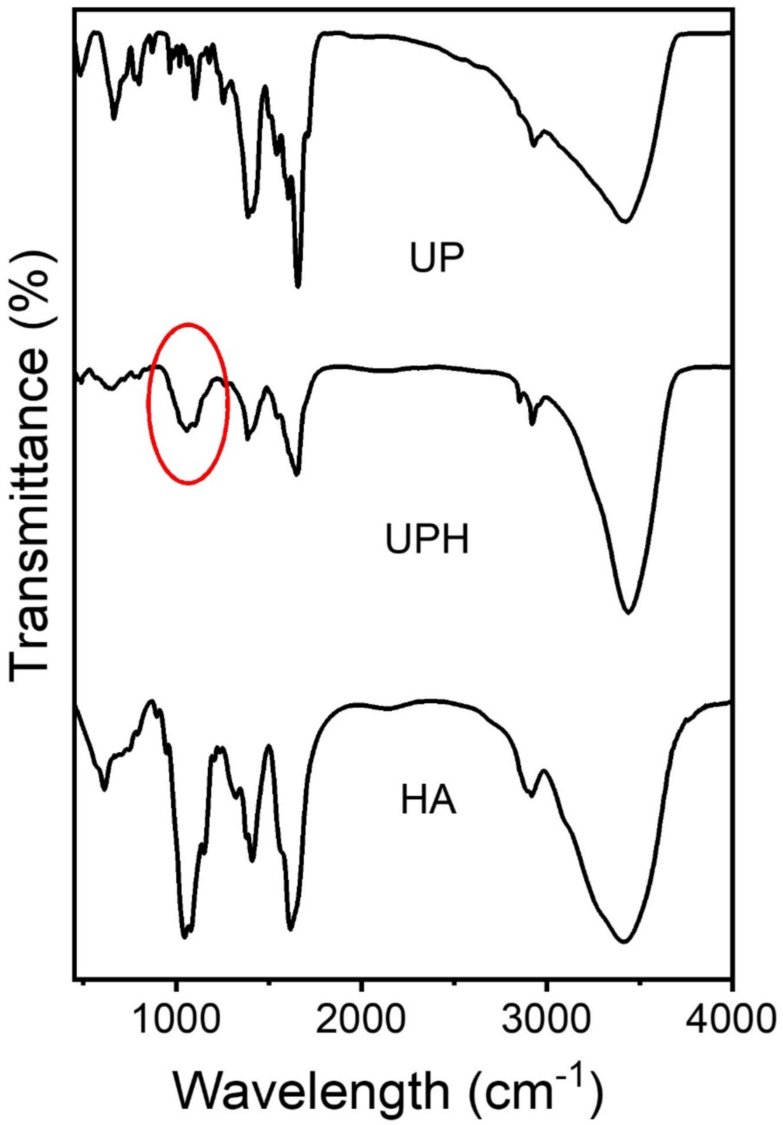

Figure S6. Infrared spectrum of different samples. The red circles represent the characteristic peaks of HA.

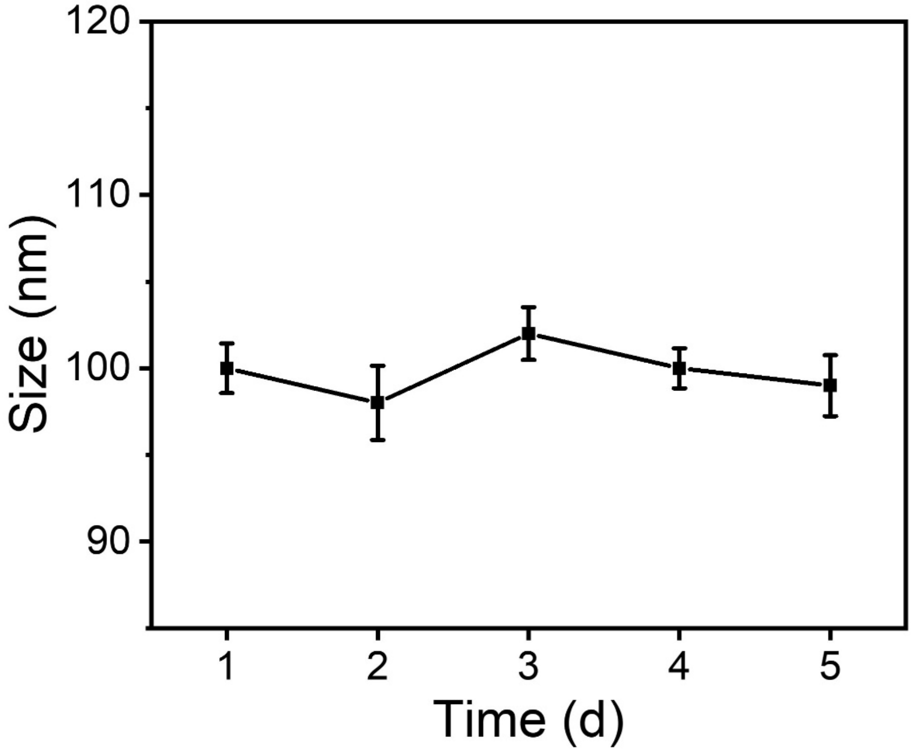

Figure S7. Size of UPH nanoparticles in PBS buffer over time.

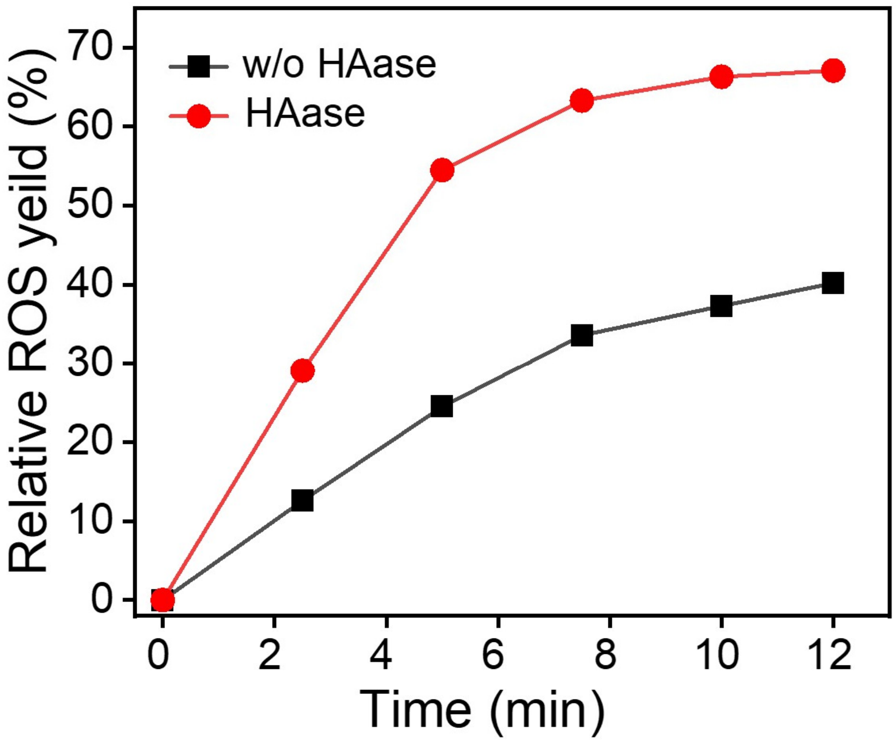

Figure S8. ROS yield of UPH nanoparticles with/without HAase.

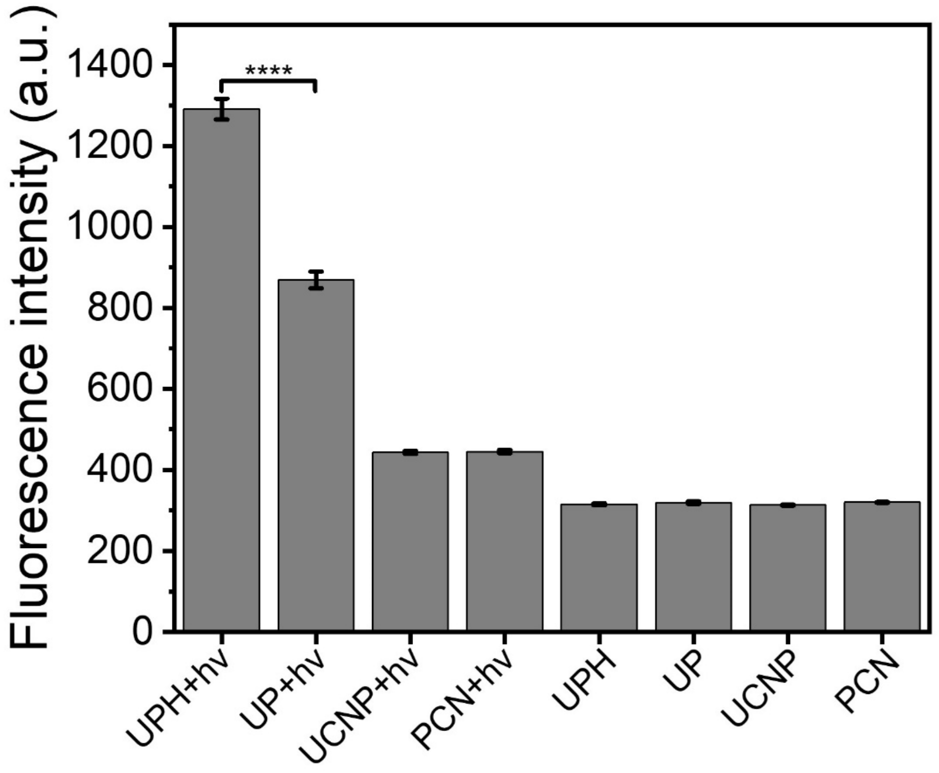

Figure S9. Statistical analysis data of cellular ROS fluorescence by CLSM. ****$P < 0.0001$

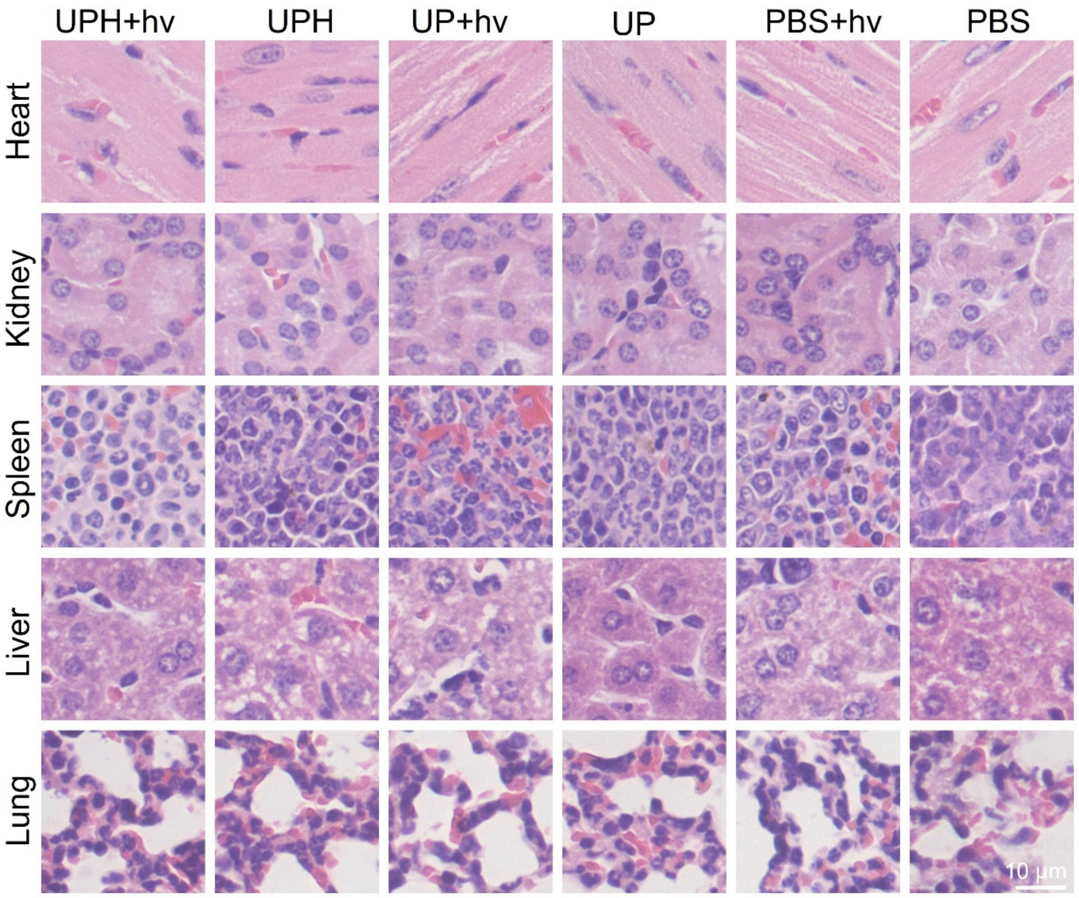

Figure S10. H&E staining analysis of main organs after different treatments for evaluation of biocompatibility.
